# Angiotensin (1–7) Expressing Probiotic as a Potential Treatment for Dementia

**DOI:** 10.3389/fragi.2021.629164

**Published:** 2021-03-30

**Authors:** Abbi R. Hernandez, Anisha Banerjee, Christy S. Carter, Thomas W. Buford

**Affiliations:** 1 Division of Gerontology, Geriatrics, and Palliative Care, Department of Medicine, University of Alabama at Birmingham, Birmingham, AL, United States,; 2 UAB Center for Exercise Medicine, University of Alabama at Birmingham, Birmingham, AL, United States,; 3 Integrative Center for Aging Research, University of Alabama at Birmingham, Birmingham, AL, United States,; 4 Nathan Shock Center, University of Alabama at Birmingham, Birmingham, AL, United States

**Keywords:** Alzheimer’s disease, gut-brain axis, renin angiotensin system, genetically modified probiotic, microbiome, Cognition, MAS1 Receptor, age-related memory loss

## Abstract

Increasing life expectancies are unfortunately accompanied by increased prevalence of Alzheimer’s disease (AD). Regrettably, there are no current therapeutic options capable of preventing or treating AD. We review here data indicating that AD is accompanied by gut dysbiosis and impaired renin angiotensin system (RAS) function. Therefore, we propose the potential utility of an intervention targeting both the gut microbiome and RAS as both are heavily involved in proper CNS function. One potential approach which our group is currently exploring is the use of genetically-modified probiotics (GMPs) to deliver therapeutic compounds. In this review, we specifically highlight the potential utility of utilizing a GMP to deliver Angiotensin (1–7), a beneficial component of the renin-angiotensin system with relevant functions in circulation as well as locally in the gut and brain.

## INTRODUCTION

Life expectancy continues to increase in developed countries worldwide, with persons aged >65 years representing the fastest growing segment of the population (Federal Interagency Forum on Aging-Related Statistics, 2006). Among the most pressing public health issues related to aging is the preservation of cognitive function and the prevention/treatment of dementia (Insel et al., 2013). Over 50 million people suffer from dementia worldwide, of which 30–35 million have Alzheimer’s disease (AD) (World Health Organization, 2019). In addition to the tremendous impact on affected patients and their families, cognitive decline is estimated to cost the healthcare system of the United States between $200–300 billion annually (Hurd et al., 2013). Thus, efficacious treatments for preventing or treating dementia are desperately needed, but to date few therapeutic strategies have shown significant promise. In particular, clinical trials in AD have proven challenging and there are no currently approved therapies which directly target AD pathology. As a result, many have called for fresh approaches to dementia treatment and prevention (Carrillo and Vellas, 2013; Zheng et al., 2015; Fish et al., 2019)—in line with major U.S. National Institutes of Health (NIH) initiatives (Insel et al., 2013). Given the substantial challenge in identifying efficacious treatments, novel research in this area is urgently needed.

To date, most approaches to treat or prevent AD have directly targeted the brain/central nervous system (CNS). In line with the need for fresh approaches, it is possible that alternative strategies are needed that target other physiologic systems beyond the CNS. One prominent possibility is targeting the gut microbiota—the microorganisms which compose the complex microenvironment of the human intestinal tract—given their known roles in neural communication (Hu et al., 2016; Alkasir et al., 2017; Proctor et al., 2017). For instance, studies in neurodegenerative diseases including Parkinson’s (Sampson et al., 2016) and Multiple Sclerosis (Berer et al., 2011) have demonstrated gut-neural communication related to disease pathology. Notably, advanced age is associated with changes to both the composition and stability of gut microbiota (Buford, 2017), and dysregulated microbiota (i.e., dysbiosis) has been is associated with cognitive impairment (Cattaneo et al., 2017; Sun et al., 2019). Multiple studies have also indicated that dysbiosis in frail seniors is associated with chronic systemic inflammation (Buford, 2017)—a hallmark of aging and biomarker of numerous age-related conditions including AD. We reviewed the potential of the gut microbiome as a therapeutic target for age-related cognitive decline (Sun et al., 2019), but original studies remain sparse which target the gut microbiome as a therapeutic site of intervention for AD.

One potential approach which our group is currently exploring is the use of genetically-modified probiotics (GMPs) to deliver therapeutic compounds. GMPs allow for the oral delivery of therapeutic compounds by embedding the compound of interest within a bacterial (i.e., probiotic) strain safe for human consumption. Indeed, several authors have purported the potential benefits and therapeutic applications of GMPs—particularly those from lactic acid bacteria (Bermúdez-Humarán et al., 2013; LeBlanc et al., 2013; Cano-Garrido et al., 2015; Börner et al., 2019; Plavec and Berlec, 2019). Major benefits of this approach to drug delivery include (1) typical inherent benefits of the bacteria itself, (2) ease of production, (3) ability for oral administration due to the ability of the bacteria to survive gut digestive processes, and (4) ability to influence both systemic and mucosal immune responses.

In fact, GMPs have already been successfully used to treat Crohn’s disease and ulcerative colitis (Barra et al., 2020), phenylketonuria (Durrer et al., 2017), and irritable bowel syndrome (Shigemori and Shimosato, 2017), among other conditions (Taylor and Lamont, 2005). However, its use in the targeting of cognitive impairment is understudied, though the potential benefits are numerous, as outlined herein. In this review, we specifically highlight the potential utility of utilizing a GMP to deliver Angiotensin 1–7 [Ang(1–7)], a beneficial component of the renin-angiotensin system (RAS) with relevant functions in not only the circulation, but also relevant tissues including the intestines and brain. Though there is ample evidence that this compound is beneficial in many ways, systemic infusion of Ang (1,7) itself is not a feasible option, as it’s half-life would require a near constant infusion (Fyhrquist et al., 1976). Our previous work has demonstrated that oral administration of Ang (1,7)-expressing probiotic [*Lactobacillus paracasei* (LP) modified to express Ang (1–7); Ang (1–7) GMP] not only increased circulating levels of Ang (1,7), it significantly altered several components of the gut-brain-axis in ways that direct systemic Ang (1,7) administration did not (Carter et al., 2019; Buford et al., 2020). Moreover, oral administration of Ang (1,7) GMP significantly altered microbial richness and diversity within the gut, increased circulating Ang (1,7), decreased circulating AngII, and altered several metabolites of tryptophan metabolism in serum, demonstrating its ability to affect changes beyond those observed within the digestive tract where it was placed. However, these data provide evidence that it is not directly Ang (1,7) itself improving peripheral health, as systemic injections did not have similar effects, but perhaps it’s ability to improve gut health that then has further reaching secondary effects on other organ systems.

## THE GUT MICROBIOME

Once considered the “forgotten organ,” the intestinal microbiota have received a tremendous amount of attention in recent years for their role in human health and disease. Indeed, gut dysbiosis has been implicated in the development of pulmonary (Bruzzese et al., 2014), neurologic (Berer et al., 2011; Hu et al., 2016; Sampson et al., 2016), cardiometabolic, and musculoskeletal conditions (Buford, 2017; Sun et al., 2019).

Notably, the majority of the body’s constitutive immune function is dedicated to maintaining homeostasis with the microbiota—evidenced by the fact that 70% of the body’s lymphocytes reside in the gut-associated lymphoid tissue (Collins et al., 2012). Once thought to contribute primarily to allergic and/or inflammatory intestinal disorders, the gut microbiota are now known to communicate with organs far from the intestine (Belkaid and Naik, 2013; Cohen et al., 2017). In fact, it is now well-established that a complex system of communication exists between the gut microbiota and the CNS.

**FIGURE 1 |:**
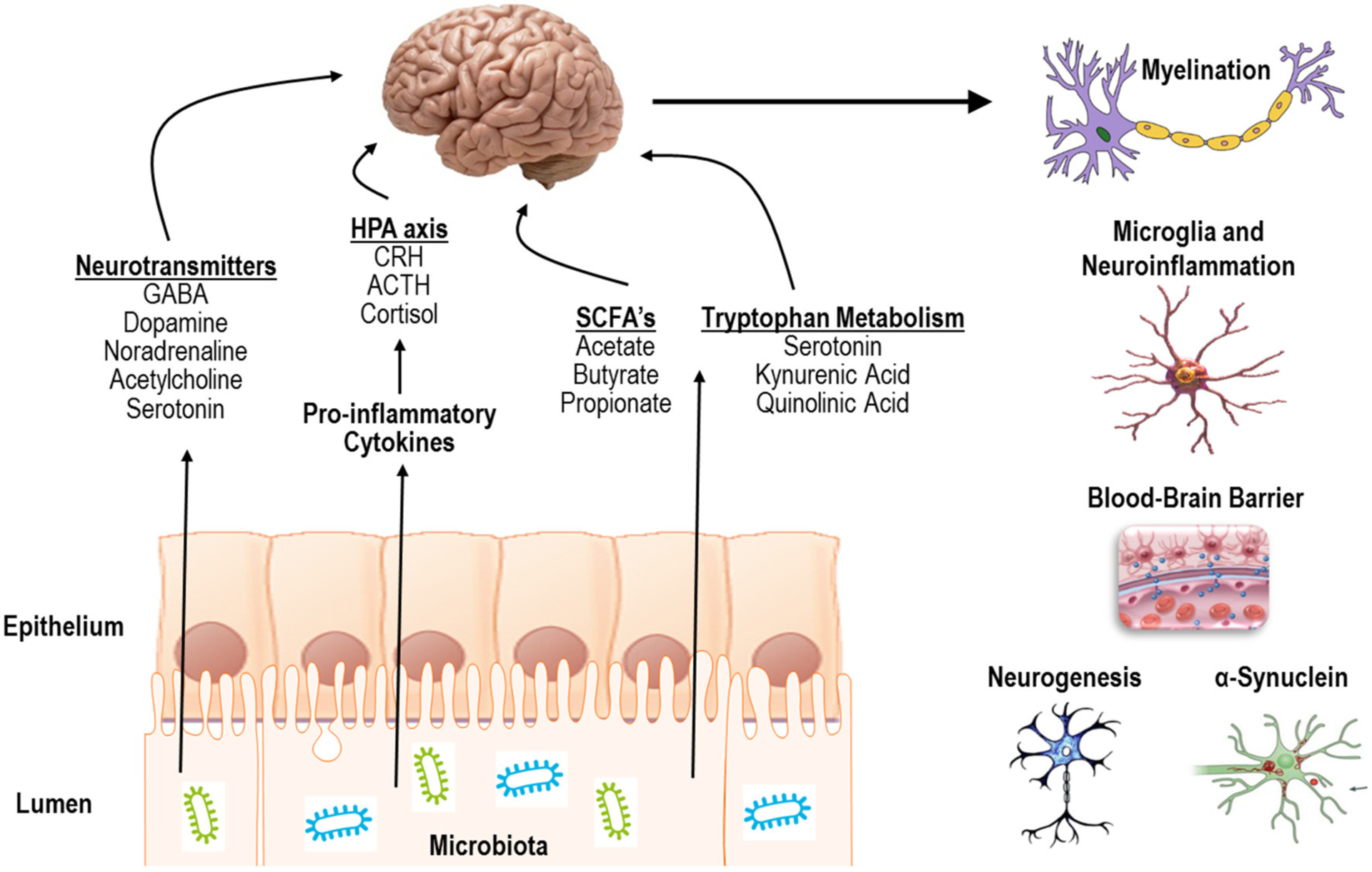
Mechanisms of action and processes affected by communication of the gut microbiota with the CNS.

Indeed, the gut microbiota can communicate with the CNS in several different ways including (1) release of pro-inflammatory cytokines to activate the hypothalamic pituitary adrenal (HPA) axis or directly impact CNS immune activity, (2) production of short chain fatty-acids, (3) release of neurotransmitters, or (4) by modulating tryptophan metabolism and downstream metabolites (Figure 1, left) (Sharon et al., 2016; Hampton, 2017; Kennedy et al., 2017; Strandwitz, 2018; Fülling et al., 2019). In summary, the literature to date has demonstrated a critical impact of the microbiota on several CNS processes including myelination, neuroinflammation, regulation of blood-brain barrier integrity, as well as regulation of neurogenesis and accumulation of α-synuclein (Figure 1, right).

Human studies have reported advanced age is associated with gut dysbiosis, which was subsequently associated with cognitive impairment (Cattaneo et al., 2017). More recently, at least one human study reported microbiome alterations in patients with AD (Vogt et al., 2017), while a study in *Drosophila* demonstrated that indeed gut dysbiosis accelerates the progression of AD in the fly model of AD (Wu et al., 2017). Several rodent models of AD have also reported gut microbiome alterations (Brandscheid et al., 2017; Minter et al., 2017; Shen et al., 2017; Zhang et al., 2017; Bäuerl et al., 2018; Chen et al., 2020). These data, coupled with similar findings from other neurodegenerative conditions (Berer et al., 2011; Sampson et al., 2016), suggest that the gut microbiome may be an important target for preserving brain health and combating AD. In line with this concept, at least two small studies have explored the potential of delivering probiotics to treat AD (Akbari et al., 2016; Leblhuber et al., 2018). These studies look to leverage the established actions of probiotics on gut microflora composition, the colon mucus barrier, and systemic immunity to treat AD. Another approach—which we discuss here—is to capitalize upon these inherent benefits of probiotics while simultaneously delivering another therapeutic compound.

Indeed, GMPs have been purported as a highly promising strategy for treating disease (Steidler, 2003; Syvanen, 2003; Paton et al., 2012) as they offer an efficacious method to deliver drugs or other therapeutic proteins with precision and a higher degree of site specificity than conventional drug regimens (Kumar M. et al., 2016). Mucosal administration of therapeutic molecules offers several important advantages over systemic delivery including the possibility of oral administration, fewer secondary effects, and the ability to simultaneously modulate both systemic and mucosal immune responses (Börner et al., 2019). To date, GMPs have been utilized to treat a limited number of conditions including inflammatory bowel disease (Steidler et al., 2003) and hypertension (Yang et al., 2015). Still, the scientific community is in the infancy of exploring the potential for GMPs. To our knowledge, no study to date has leveraged the GMP approach—Ang (1–7) or otherwise—to improve cognition. Thus, a tremendous opportunity exists to leverage existing knowledge and advances in technology within this space.

## DEMENTIA TYPES/GROWING CONCERNS

Various forms of dementia affect more than 70% of individuals over the age of 70 and cost the United States ~$200 billion in 2010 (Hurd et al., 2013). Furthermore, dementia severely decreases quality of life for both patients and caregivers, and neurologic conditions are the third leading cause of disability globally. While cognitive slowing and difficulty multitasking are hallmarks of normal aging, dementias—including mild cognitive impairment (MCI) and Alzheimer’s disease—result in far greater cognitive impairments.

Often thought of as an intermediary step in the progression of dementia is a disease state known as mild cognitive impairment (MCI). While patients with MCI demonstrate impaired cognitive processes beyond those of normal aging, they are not as severe as individuals with more advanced neurodegenerative disease states. While individuals with MCI are more likely to develop Alzheimer’s disease (AD) and other forms of more advanced dementia than age matched controls (Boyle et al., 2006), there are distinct differences in the microbiome profiles of healthy aged control subjects, those with MCI and those with AD (Liu et al., 2019).

Alzheimer’s disease (AD), one of the most prevalent forms of dementia, currently affects 5.8 million people in the United States and is the sixth overall leading cause of death. Typical AD pathology includes extra cellular deposits of amyloid β-protein (Aβ) and neurofibrillary tangles of hyperphosphorylated tau (Selkoe, 2001), though more recent pathological theories of disease etiology include metabolic factors (de la Monte and Wands, 2008). Inclusion of metabolic factors may be critical to preclinical models and therapeutic interventions for AD, as 80% of people with AD also have metabolic deficits, usually in the form of type II diabetes or insulin resistance (Janson et al., 2004). In addition to neuropathology, chronic inflammation and disruption of immunoregulatory functions precede AD-related cognitive decline (Schmidt et al., 2002). Furthermore, vascular contributions to cognitive impairment in dementia (VCID) likely plays a role in AD etiology (Jagust, 2001; de la Torre, 2004) and there is evidence that the microbiome plays a role in vascular health (Grant and Jönsson, 2019). These links between peripheral health and brain pathology suggest the gut-brain-axis could be an important factor in healthy brain aging and dementias, as each of these areas of peripheral health are influences by gut health and functioning (Honda and Littman, 2012; Nieuwdorp et al., 2014; O’Mahony et al., 2015; Grant and Jönsson, 2019; Chen et al., 2020; Ragonnaud and Biragyn, 2021).

Indeed, emerging evidence indicates individuals with AD demonstrate an altered microbiome profile with decreased diversity beyond that of age-matched controls (Vogt et al., 2017). Although only two studies to date have implemented a probiotic approach to treatment in human AD patients, results have been promising. For example, probiotic administration for 12 weeks was reported to improve Mini-Mental State Examination (MMSE) scores as well as several markers of metabolic health including insulin sensitivity in AD patients relative to the placebo group (30 participants/group) (Akbari et al., 2016). In an additional study, multispecies probiotic supplementation for 28 days significantly altered serum kynurenine levels, which may influence nervous system function (Leblhuber et al., 2018).

Though not an overt form of dementia, Parkinson’s disease (PD) is another neurodegenerative disease with potential cognitive impacts and affects ~1-million individuals in the United States alone (Marras et al., 2018). Interestingly, one of the earliest complaints in individuals who eventually receive a PD diagnosis is constipation, and PD is associated with slow colonic transit (Sakakibara et al., 2003), increased intestinal permeability (Schwiertz et al., 2018) and by alterations in the RAS (Allen et al., 1992). As with other conditions affecting neurological function, PD patients demonstrate specific alterations in gut microbiome (Tan et al., 2014). While probiotics have been utilized for a while for the treatment of constipation in PD patients (Cassani et al., 2011), more recent work has investigated the efficacy of probiotic treatment for movement and metabolic parameters in PD (Tamtaji et al., 2019). In this randomized double-blind trial, probiotic supplementation for 12 weeks improved motor symptoms as well as metabolic profiles. Similar motor improvements, as well as cognitive benefits, have been noted in a mouse model of PD (Perez-Pardo et al., 2017). These data from PD further suggest that altering the gut microbiome may influence cognitive outcomes in other neurodegenerative conditions, such as AD.

## ANGIOTENSIN (1–7), THE CNS AND POTENTIAL MECHANISMS THROUGH WHICH ANG (1–7) GMP MAY INFLUENCE COGNITIVE FUNCTION AND PATHOLOGY

**FIGURE 2 |:**
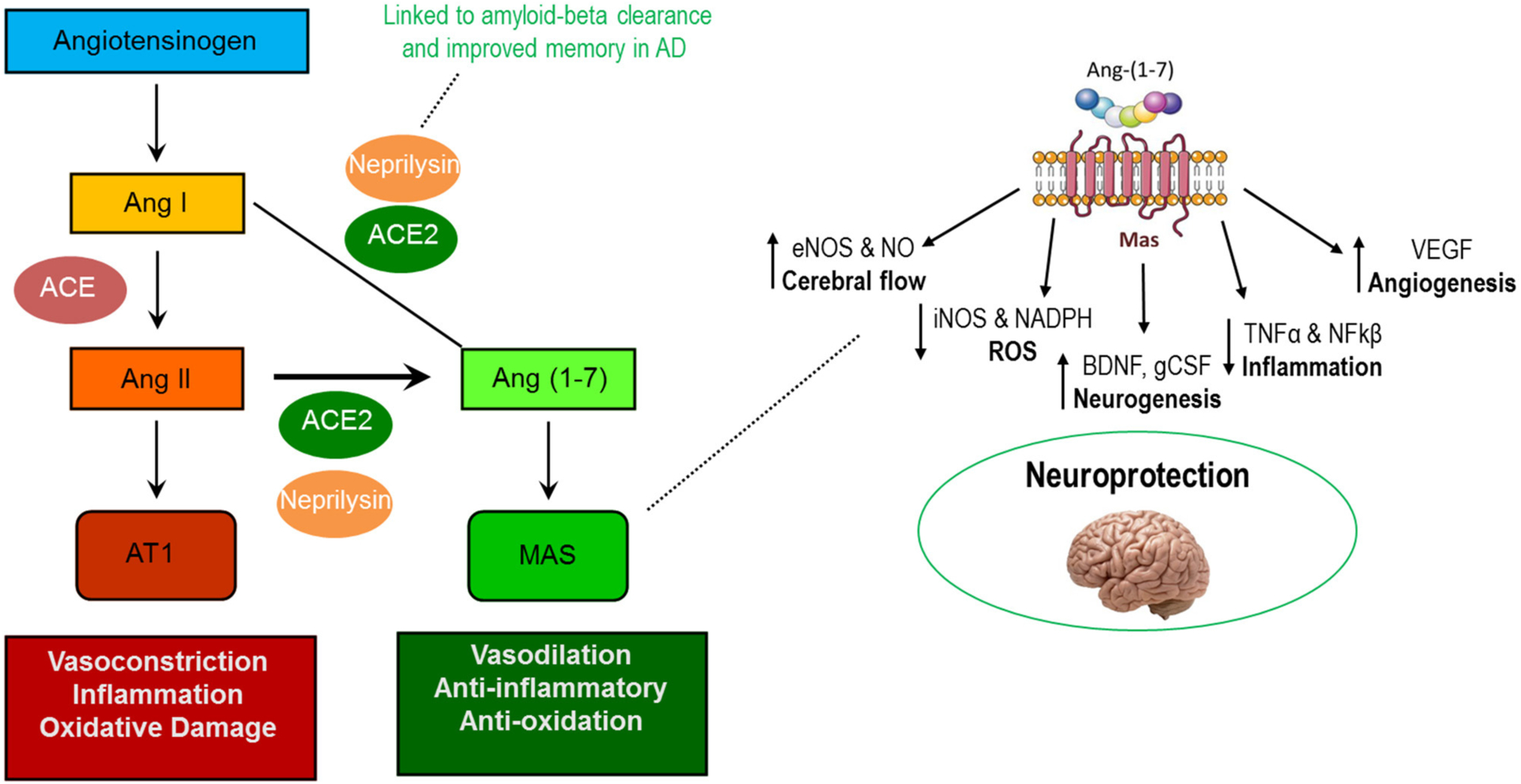
Actions of the AT1R-mediated and MasR-mediated axes of the renin-angiotensin system (left) and known neuroprotective actions of Ang (1–7) in the central nervous system (right).

In recent years a number of pleiotropic effects have been ascribed to the renin-angiotensin system (RAS) that extend beyond lowering blood pressure (Sica, 2011; Zoccali and Mallamaci, 2014). As we reviewed (Simon et al., 2015), beneficial impacts of the RAS may be induced not only by antagonizing the vasoconstrictive actions of angiotensin II (AngII) binding to the AT_1_ receptor (AT_1_R), but also by the more recently discovered Ang (1–7) axis whereby the binding of Ang (1–7) to the Mas (AT_7_) receptor promotes several beneficial actions (Figure 2, left). However, the vascular effects of RAS-affecting compounds should not be completely ignored, as individuals with AD often present with underlying vascular impairment (Jagust, 2001; de la Torre, 2004).

Among the purported benefits of the Ang (1–7) axis is improved brain health. Components of the axis are found throughout the central nervous system (CNS) including within neurons, astrocytes, cerebral arteries, and various brain regulatory centers including the paraventricular nucleus (PVN) and hypothalamus (Hamming et al., 2004; Gallagher et al., 2006; Becker et al., 2007; Doobay et al., 2007; Guo et al., 2010). Notably, Ang (1–7) stimulates numerous molecular pathways responsible for beneficial adaptations which could contribute to improved cognitive function including increased (1) endothelial nitric oxide synthase (eNOS), (2) brain-derived neurotrophic factor (BDNF), and (3) vascular endothelial growth factor (VEGF) as well as reduced production of reactive oxygen species and pro-inflammatory cytokines (Figure 2, right) (Passos-Silva et al., 2013; Jiang et al., 2014; Zheng et al., 2014; Wang et al., 2016; Kamel et al., 2018).

In line with these actions, evidence from both human and rodent models indicates that Ang (1–7) axis modulation benefits multiple aspects of cognition in several models of cognitive dysfunction, including AD (Kehoe et al., 2016; Wang et al., 2016; Hay et al., 2017, 2019; Kangussu et al., 2017; Kamel et al., 2018). Notably, animal studies have shown remarkable consistency across both loss and gain of function genetic models as well as via pharmacologic stimulation of the Ang (1–7) axis. Moreover, at least two human studies have proposed both systemic (Jiang et al., 2016) and brain (via post-mortem study; Kehoe et al., 2016) Ang (1–7) axis activity as biomarkers of AD pathology including association with amyloid-β and tau. Additionally, the plasma membrane glycoprotein neprilysin—involved in endogenous production of Ang (1–7)—reduces amyloid-β and improves memory in mice and drosophila (Hüttenrauch et al., 2015; Turrel et al., 2017). These data suggest that Ang (1–7) administration could hold promise for preserving cognitive function in late life as well as in combating AD.

Currently, recombinant Ang (1–7) is being utilized in clinical trials for clinically-urgent conditions including inoperable tumors, breast cancer, mitigation of chemotherapy induced bone marrow toxicity, and wound healing (Source: www.clinicaltrials.gov). However, systemic delivery of Ang (1–7) typically requires burdensome infusions and/or repeated injections as well as the expensive and cumbersome production process for the drug. Thus, a different formulation—particularly one which could be administered orally–could provide practical benefits to patients from both a cost and ease of use standpoint. In line with this reasoning, our previous work has demonstrated that orally delivered Ang (1,7) GMP influences far beyond the gut, including altering levels of neurotransmitters and components of the RAS (Buford et al., 2020).

## ANGIOTENSIN (1–7) AND THE GUT

Components of the Ang (1–7) axis are also present throughout the gut including the small intestinal brush border, muscularis mucosa, and propria, as well as microvascular endothelium and vascular smooth muscle cells (Hamming et al., 2004). In fact, the highest tissue concentrations of mRNA for ACE2—the enzyme responsible for producing Ang (1–7) endogenously—are found in the terminal ileum, duodenum, and colon (Tipnis et al., 2000; Harmer et al., 2002). As a result, ACE2 has received attention for its connection to the gut microbiome (Perlot and Penninger, 2013; Cole-Jeffrey et al., 2015; Raizada et al., 2017).

**FIGURE 3 |:**
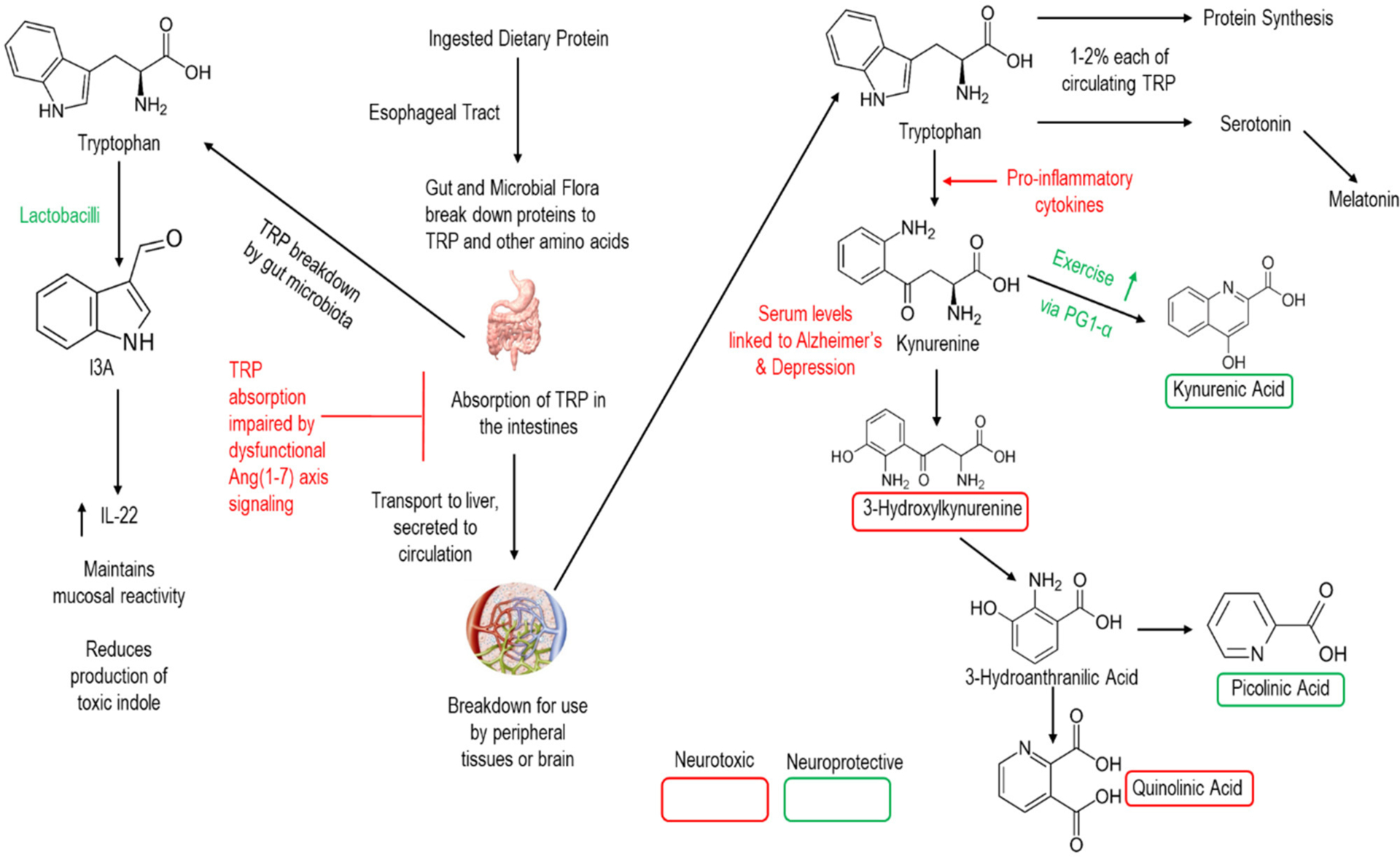
Simplified schematic of human breakdown of tryptophan and influences on CNS signaling. Note that lactobacilli (the bacterial strain in our GMP) and the Ang (1–7) axis are active in this process.

One critical link of gut Ang (1–7) to CNS function is in the metabolism of the essential amino acid tryptophan (Figure 3). Under normal conditions, gut microbiota directly metabolize ~5% of ingested tryptophan into various bioactive compounds related to the intercellular signaling molecule indole. Indole derivatives can have beneficial or toxic effects, and the regulation of derivative production is dependent upon the composition of the intestinal microbiota.

The remaining (~95%) tryptophan is then metabolized by the human host and absorbed in the intestines for transport to the liver and release into the circulation. Once into the circulation, a minority of tryptophan is broken to support protein synthesis and the production of the neurotransmitter serotonin. Meanwhile the vast majority of tryptophan is metabolized to the neuro-regulatory kynurenine pathway—which has been proposed a key link between gut dysbiosis and various neurologic conditions including AD (Widner et al., 2000; Gulaj et al., 2010). Indeed, downstream metabolites of kynurenine are key CNS signaling molecules providing either neuroprotective or neurotoxic effects to the brain. Notably, members of the genus *Lactobacilli* promote the production of Indole-3-carboxaldehyde (I3A) which promotes the production of interleukin 22 and the maintenance of mucosal reactivity (Zhang and Davies, 2016). Meanwhile, ACE2 is essential to intestinal absorption of tryptophan (Hashimoto et al., 2012; Singer et al., 2012) as well as in regulating intestinal immune function, ecology of the gut microbiome, and attenuating intestinal inflammation suffered in response to epithelial damage (Singer et al., 2012). Thus, both *Lactobacilli* and Ang (1–7) hold promise for altering these neuro-regulatory pathways.

We recently published data from a short-term dosing study indicating that the Ang (1–7) GMP reduced neuroinflammatory gene expression in the pre-frontal cortex while also increasing circulating concentrations of neuroregulatory compounds picolinic acid and serotonin in older F344/BN rats (Buford et al., 2020). Our findings also indicated that the use of an Ang (1–7)-expressing GMP was more efficacious than subcutaneous injection of a synthetic Ang (1–7) peptide, further suggesting the possible utility of the GMP-based approach. Still, these studies were short-term in an animal model without overt dementia, thus continued exploration is needed in this area to document the full potential utility of this intervention in the preservation of cognitive function—particularly in models of AD.

## EARLY STUDIES OF RAS-AFFECTING COMPOUNDS IN PRE-CLINICAL AND CLINICAL AD

Much of the previous work manipulating the RAS in preclinical animal models (see Table 1) has utilized one of two categories of drugs, angiotensin receptor blockers (ARBs) and angiotensin converting enzyme inhibitors (ACEI). While both categories of drugs target the same biochemical pathway, ARBs work by blocking AngII from binding to its receptor, AT_1_R, while ACE inhibitors block the conversion of AngI to AngII (Wright et al., 2013). Both ARBs and ACEIs shift activity of the RAS system away from the AT1-mediated axis toward the Mas-mediated axis (see Figure 2), and therefore may be efficacious in protecting the neurophysiological milieu from various cognitive deficits (see Figures 2, 3).

ARB utilization in preclinical animal models of AD has demonstrated both neuro- and vaso-protective effects when delivered chronically. Intranasal administration of candesartan, a particularly potent and blood brain barrier-permeable ARB, for 8 weeks in a 5XFAD mouse model of familial AD was anti-inflammatory, as it reduced hippocampal microglial activation and Aβ pathology (Torika et al., 2018). However, an amyloid precursor protein (APP) mouse model utilizing candesartan did not observe altered Aβ pathology (Trigiani et al., 2018), suggesting not all AD preclinical models respond similarly to ARBs. While there were partial memory enhancing effects of the ARB losartan in APP mutated mice, these effects were ameliorated by selective blockade of angiotensin IV (AngIV) at its receptor (AT4R), implicating the angiotensin IV/AT4R cascade as a promising candidate for AD intervention (Royea et al., 2017). Because telmisartan is also a peroxisome proliferator-activated receptor (PPAR)-γ agonist capable of exerting anti-inflammatory effects in neurons, these results indicate it may be a particularly useful ARB for the treatment of AD through its combined PPAR-γ and AT_1_R blockades, resulting in attenuated Aβ deposition (Mogi et al., 2008) and improved cognitive outcomes (Tsukuda et al., 2009). Other ARBS, including valsartan and eprosartan, demonstrated no effect on Aβ or APP pathology (Ferrington et al., 2011, 2012), though results are mixed (Wang et al., 2007), so caution should be taken when selecting an ARB to utilize in preclinical AD trials.

Several studies have also demonstrated great efficacy of ACEIs in pre-clinical AD rodent models. In particular, centrally-active ACEIs, such as perindopril, prevent AD-associated cognitive decline, hippocampal microglial & astrocytic activation, and oxidative stress in these animal models utilizing intracerebroventricular Aβ injections, even after as little as 1–7 days of treatment (Yamada et al., 2010; Dong et al., 2011). In contrast, ACEIs that are not centrally active, such as imidapril and enalapril, are ineffective under these same conditions (Yamada et al., 2010; Dong et al., 2011). Longer term administration of perindopril or captopril (also centrally active) delivered intranasally can also improve amyloid burden (Torika et al., 2016), demonstrating feasibility of delivering these compounds peripherally rather than intracerebrally. However, orally delivered captopril did not influence Aβ or APP pathology after 2 (Ferrington et al., 2011) or 6 (Ferrington et al., 2012) months of administration in 3xTgAD mice. Notably, the aforementioned ARB and ACEI studies all utilized young adult animal models of AD. However, it is imperative that aged animals be included in future investigations of the effects of RAS-affecting compounds in AD-related research, as translational studies will not be conducted in young humans, but within the aged population naturally afflicted by this disease state.

The rationale for targeting RAS through a GMP includes not only the aforementioned promising effects of ACE-affecting drugs, but also the interaction between AD and the gut microbiome as demonstrated by animal models. Not only is the gut microbiome of APP transgenic mice significantly shifted, but germ free APP transgenic mice demonstrate significantly reduced Aβ pathology (Harach et al., 2017; Minter et al., 2017). Similarly, overexpression of α-synuclein, which normally results in Parkinson’s-like phenotypes, in germ free mice result in significantly reduced pathological and physical burden (Sampson et al., 2016). Treatment with antibiotics capable of inducing prolonged shifts in gut microbiota also influences neuroinflammation and amyloidosis in a APP/PS1 mouse model (Minter et al., 2016), further suggesting a link between microbiota and AD-related pathology. Work by Kumar et al. demonstrates that there is a potential protective, antimicrobial role for Aβ pathology, thus demonstrating a possible microbial basis for triggering Aβ pathology (Kumar D. K. V. et al., 2016). All together, these data demonstrate that translating RAS-affecting manipulations to humans must not only be easily feasible, but may be particularly effective when it targets the gut. Additional evidence for the link between the RAS and AD is reviewed in Kehoe (2018).

On the other hand, human studies on the influence of angiotensin on AD are sparse, positive outcomes have been reported using ACE inhibitors and ARBs clinically (Quitterer and AbdAlla, 2020). ACEI use is capable of slowing cognitive decline in individuals diagnosed with AD (O’Caoimh et al., 2014), particularly within the first 6 months (Gao et al., 2013). Continuous, or even intermittent, ACEI use over 4 years by individuals with AD lead to significantly less decline in MMSE scores relative to those who had never used ACEIs, including individuals on other antihypertensive drugs (Soto et al., 2013). In a group of individuals with both AD and hypertension, prolonged ARB administration increased Aβ1–42, decreased IL-1β and TNF-α and improved cognitive scores relative to controls administered a calcium channel blocker for the same duration (Wei et al., 2012). Among veterans with cardiovascular disease, individuals prescribed an ARB that developed AD were slower to require assisted living than AD control patients (Li et al., 2010). Furthermore, a combination of diuretics, calcium channel blockers and ARBs slow cognitive decline (Hu et al., 2018), indicating the potential for synergistic interventions targeting multiple systems. In fact, there is evidence that ARBs may be more effective than ACEIs in regard to cognitive outcomes, independent of their ability to lower blood pressure (Hajjar et al., 2020). However, the optimal timeframe of delivery for ARBs and ACEIs is unclear. While these studies include examples of slowing of already present cognitive decline, whether earlier and/or longer term administration of either class of drug could be preventative of AD and other dementias is unknown, but is worth considering in future endeavors.

## POTENTIAL MECHANISMS OF ANG (1,7) GMP EFFICACY

The aforementioned preclinical and clinical studies demonstrate the potential of RAS-affecting compounds for the treatment of AD and dementia. However, a probiotic with not only the ability to target these systems, but to restore gut dysbiosis, such as the Ang (1,7)-expressing *Lactobacillus paracasei* (LP), may provide a better way to target AD and age-related dementia. Moreover, the delivery of therapeutic compounds through probiotics is particularly useful due to their ability to survive gastric acids and bile, allowing them to reach the intestinal target. The specific GMP discussed here utilized LP as the live vector for oral delivery. The LP was then modified to express Ang (1,7) as a secreted fusion protein utilizing cholera toxin subunit B (the non-toxic subunit; CTB), which functions as a trans-epithelial carrier, allowing for uptake into circulation (Verma et al., 2020). Although there is little evidence that oligopeptides, other than di- and tripeptides, can normally cross the mucosal border (Miner-Williams et al., 2014), CTB is able to facilitate transmucosal transport through GM1 receptor mediated endocytosis (Baldauf et al., 2015; Verma et al., 2020). Once this conjoined molecule is secreted into the circulatory system, the CTB is separated from the Ang (1,7) through a furin cleavage site. Expression of Ang (1,7) itself, within several tissues and in serum, has previously been confirmed in mice and rats (Carter et al., 2019; Verma et al., 2020). It is likely that the effects on peripheral and CNS health are synergistic, as they are densely interconnected. There are several mechanisms by which Ang (1,7) GMP may be beneficial for AD, including, but not limited to (1) improved vascular health through the RAS, (2) improved gut dysbiosis, (3) altered neurotransmitter metabolism within the gut and (4) improved glucose metabolism.

### Improved Vascular Health

First and foremost, the vascular effects of any RAS-affecting compound should be considered. Direct application of the Ang (1,7) GMP into the gut is able to get into circulation (Verma et al., 2019; Buford et al., 2020), where it may then mimic the effects of ARBs and ACEIs through the downregulation of the vasoconstrictive arm of the RAS. The resulting vasodilation may lead to an improvement in AD-related impairments, as many individuals with AD also experience impaired cardiovascular function (Jin et al., 2017). Therefore, this RAS-targeting probiotic may rescue or reduce the cognitive phenotype associated with vascular dementia. However, differentiation between improved neural function and vascular function has yet to be established, though the two are likely synergistically improving outcomes as peripheral and cognitive health are strongly and reciprocally linked.

### Repairing Gut Dysbiosis

It is conceivable that the actions of Ang (1,7) GMP work to improve CNS function through the targeting other peripheral tissues, rather than through targeting the brain directly. Impaired gut function can impair brain health and cognitive functioning, and may result in neurodegenerative conditions (Berer et al., 2011; Gareau, 2014; Sampson et al., 2016; Cattaneo et al., 2017), so it is plausible that the restoration of gut health would ameliorate these deficits. The probiotic itself, *Lactobacillus paracasei*, may be able to restore dysbiosis (Kim et al., 2019), which is present in individuals with AD. Both the ACE2/Ang (1,7)/MAS1 axis of the RAS and the probiotic help to reduce inflammation (Gaddam et al., 2014; Wang et al., 2020), which may then decrease gut leakiness and prevent the ability of inflammatory markers and amyloid proteins from escaping into circulation (Pistollato et al., 2016).

### Altered Neurotransmitter Metabolism

The intestinal tract is an integral part of many bodily systems, including the nervous system through the generation of many neurotransmitter molecules (Yano et al., 2015; Pistollato et al., 2016). While alterations in gut health may influence the metabolism of several neurotransmitters, RAS activity within the gut is particularly involved in tryptophan, and thus serotonin, metabolism (Figure 1, left) (Sharon et al., 2016; Hampton, 2017; Kennedy et al., 2017; Strandwitz, 2018; Fülling et al., 2019). Additionally, *lactobacillus* supplementation is capable of modulating the neurotransmitter GABA receptor expression, regulating emotional behavior in mice via the vagal nerve (Bravo et al., 2011).

### Repaired Glucose Metabolism

Unfortunately, there is both an age- (Gage et al., 1984; Rasgon et al., 2005; Goyal et al., 2017) and AD-related (Janson et al., 2004) reduction in the ability to metabolize glucose, which further impairs cognitive function in these conditions. The ACE2/Ang (1,7)/MAS1 axis of the RAS plays a significant role in glucose absorption through the intestines and activation of this pathway improves hyperglycemia in diabetic patients (Wong et al., 2007; Garg et al., 2012), Furthermore, administration of ACEIs can ameliorate type 2 diabetes (Henriksen, 2007; Favre et al., 2015) and improve obesity (Kouyama et al., 2005; Weisinger et al., 2009).

In addition to the RAS component of the GMP, the probiotic utilized to deliver the compound may improve metabolic function itself. *Lactobacillus paracasei* and other *Lactobacilli* have complex microbiome-metabolome interactions, influencing short chain fatty acid, ketone and methyl acetate concentrations (Martin et al., 2008; Vitali et al., 2010; Arora et al., 2013). Furthermore, these strains are able to increase amino acid absorption as well as beneficially alter the fermentation process (Ndagijimana et al., 2009). *Lactobacillus* itself is capable of improving glucose and insulin signaling in mouse models of obesity and diabetes (Yun et al., 2009; Naito et al., 2011), which are both common comorbidities with AD.

## POTENTIAL MODULATORS OF EFFECT NEEDING CONSIDERATION IN STUDY DESIGNS

### Biological Sex

A vast gender disparity exists in Alzheimer’s disease (AD), with two-thirds of diagnosed patients being female. Notably, this gender discrepancy is not explained by increased life expectancy as the incidence, cognitive decline, and amyloid pathology are all greater in women compared to age-matched men with AD (Barron and Pike, 2012). As a result, a significant need exists to investigate mechanisms which might contribute to these sex differences in the incidence of AD.

Notably, prior evidence indicates that male and female rats have differing circulating and renal levels Ang (1–7) and other non-classical components of the RAS (Sullivan et al., 2010). Specifically, male mice and rats both demonstrate an increased vascular response to AngII administration than their female counterparts (Tatchum-Talom et al., 2005). Additionally, female spontaneously hypertensive rats (SHRs) have decreased sensitivity to angiotensin II, demonstrating blunted hypertensive effects (Sullivan et al., 2010). This effect is mediated by the RAS system, as ACE inhibitor enalapril administration similarly reduced blood pressure levels in both sexes (Reckelhoff Jane et al., 2000).

Of particular note to those interested in studying age-related disease states such as dementia, there are sex-specific changes in serum peptidases comprising the RAS that accompany aging in humans (Fernández-Atucha et al., 2017). While aminopeptidase A (APA) was the only significantly different peptide across all men and women studied, both APA and ACE were significantly lower in male subjects when only individuals over the age of 55 were included in the analysis. Additionally, estrogen mediates neuroprotection in clinical and animal models of Parkinson’s disease, likely through inhibition of the RAS, as estrogen inhibits nigral angiotensin, mediating neuroprotection (Labandeira-Garcia et al., 2016).

Further consideration should be taken for sex differences in gut microbiota, termed the “microgenderome” by Flak et al. (2013). This concept outlined sex differences in the bidirectional interactions between gut microbiota and the body, particularly in the case of hormones and immunity. However, it should be noted that differences in microbiome composition across male and female humans are confounded by conflicting reports, with opposite changes described across differing cohorts (Vemuri et al., 2019). In any case, estrogen level in males and postmenopausal females correlate with microbiome diversity and richness, though this correlation is absent in pre-menopausal females (Flores et al., 2012). Additionally, males and females may respond to probiotic treatment differently in both humans (Lönnermark et al., 2015) and rodents (McCabe et al., 2013). Together, differences in the RAS and microbiome across male and female subjects warrant additional research and consideration in probiotic-based interventions, particularly in the case of a RAS-targeting GMP.

### Diet

Dietary intake is universally considered one of, if not the, most important factor influencing the gut microbiome. Moreover, dietary interventions including caloric restriction, altered macronutrient ratios and intermittent fasting have strong effects in promoting healthy aging. Thus, the choice of diet could have critical implications for determining if results observed with the GMP will translate to humans. However, there are several drivers of altered dietary patterns in older adults leading to decreased diversity in foods consumed, including decreased physical mobility, loss of smell or taste and slowed digestion (Britton and McLaughlin, 2013).

Alterations in macronutrient composition may be the largest driving force behind differences in gut microbial communities, as evidenced by regional differences in dietary composition from an early age, leading to demonstrable differences in gut microbiota (Voreades et al., 2014). Long-term dietary intake, particularly protein and animal fat content vs. carbohydrate content, most strongly influence gut microbiome enterotype (Wu et al., 2011). However, short term dietary changes can also rapidly influence gut microbiome composition (Wu et al., 2011; David et al., 2014). More extreme examples of altered dietary patterns, such as ketogenic (Newell et al., 2016; Olson et al., 2018; Cabrera-Mulero et al., 2019) and vegetarian or vegan diets (Tomova et al., 2019) also dramatically shift microbial composition. This is particularly relevant in the context of AD and related dementias, as both Mediterranean and ketogenic diets have been utilized in pre-clinical and clinical studies with promising results in regards to improving cognitive deficits (Solfrizzi et al., 2011; Lourida et al., 2013; McDonald and Cervenka, 2018; Rusek et al., 2019).

Although dietary composition has a large influence on microbiome composition, altered dietary patterns may have a strong influence as well. Obese individuals who restrict calorie intake demonstrate an increase in *Bacteroidetes*, significantly improving their *Bacteroidetes:Firmicutes* ratio, regardless of whether this reduction was through from altered fat or carbohydrate intake (Ley et al., 2006). Furthermore, calorie restriction improved microbial gene richness and improved metabolic parameters (Cotillard et al., 2013; Le Chatelier et al., 2013). The introduction of time restricted feeding (i.e., eating exclusively during specific times of the day) not only improves metabolic health, but improves diversity within the gut microbiome (Zarrinpar et al., 2014; van der Merwe et al., 2020).

The RAS is also heavily influenced by diet. High-salt diets can suppress systemic RAS, lowering plasma renin, angiotensin II (AngII) and aldosterone levels (He and MacGregor, 2011; Charytan and Forman, 2012). Both obesity and starvation have profound effects on angiotensinogen, the precursor to AngII (Frederich et al., 1992; Turban et al., 2002). Obese prone rats demonstrate increased angiotensin-related gene expression following 8 weeks of a high fat diet relative to lean and obese resistant rats (Boustany et al., 2004). Obesity not only negatively influences hypertension and RAS signaling, but greatly increases the risk of dementia in aged individuals (Whitmer et al., 2005; Beydoun et al., 2008). Furthermore, individuals with dementias demonstrate altered eating patterns (Morris et al., 1989; Ikeda et al., 2002; Sandilyan, 2011), perhaps enhancing age-related gut dysbiosis. Of note, several subtypes of dementias correlated with an significant increase in preference for sweet foods (Ikeda et al., 2002), which can lead to detrimental shifts in carbohydrate intake.

Beyond the direct relationship of dietary consumption patterns and altered microbial diversity, diet can have a profound influence on central nervous system function in aged subjects through alterations in energy bioavailability (Hernandez and Burke, 2018; Mujica-Parodi et al., 2020). Probiotics and fermented foods may enhance the link between the gut and brain through increased GABA, serotonin and/or BDNF (Chung et al., 2014; Kim et al., 2016).

### Physical Exercise

Physical exercise represents an important healthy aging intervention with important implications for brain health (Ahlskog et al., 2011; Buchman et al., 2012), and exercise directly modulates the gut microbiome (Mailing et al., 2019). The activity status of an organism also directly influences drug responsiveness due to differences in various hemodynamic and metabolic outcomes (Lenz et al., 2004; Lenz, 2011)—often demonstrating differing drug effects under active and sedentary states (Huffman et al., 2008; Narkar et al., 2008; Buford et al., 2012; Menzies et al., 2013). Therefore, it is possible that utilization of an Ang (1–7)-targeting GMP could be most effective when combined with physical exercise.

Exercise alone can alter the gut microbiome, as reviewed previously (Mailing et al., 2019). Specifically, exercise is able to increase the abundance of butyrate-producing taxa (Matsumoto et al., 2008; Evans et al., 2014; Kang et al., 2014). This may have interesting implications in aging individuals with or without dementia, as butyrate itself is anti-inflammatory, beneficial for metabolic health and can boost production of ketones to serve as a fuel source (Cavaleri and Bashar, 2018). This is particularly beneficial given the negative alterations in glucose utilization in aged dementia patients. However, the type of exercise may influence the gut microbiome in different ways (Allen et al., 2015) and there are conflicting data as to how certain phyla are altered in both preclinical animal models and human data (Mailing et al., 2019). However, a study utilizing the APP/PS1 transgenic mouse model of AD demonstrated significant advantage of both exercise and probiotic use (Abraham et al., 2019), strengthening the argument for physical activity and gut health in AD treatment.

Similarly, exercise can have drastic effects on the RAS. Chronic exercise not only improves systemic blood pressure, but prevents increased expression of ACE and AT_1_R in hypertensive rats—while simultaneously preventing a decrease in ACE2 and MasR expression normally observed in these animals (Agarwal et al., 2011). This study also showed that in addition to regulating the vasoconstrictor axis of the RAS, chronic exercise also attenuates inflammatory cytokine expression. Exercise training attenuates ROS within the paraventricular nucleus, a brain region involved in the RAS (Zhang et al., 2016). Additionally, exercise improves vascular sensitivity to insulin through the action of Ang (1–7) action at the MasR (Gallardo-Ortíz et al., 2020), which may be of particular relevance to individuals with insulin-related impairments including diabetes and AD.

An abundance of evidence demonstrates the potential for exercise to improve quality of life in advanced age (Mazzeo et al., 1998; Spirduso and Cronin, 2001; Rejeski et al., 2002; Courneya and Karvinen, 2007), as well as dementia (Ahlskog et al., 2011). Taken together, data from exercise studies suggest that combining a RAS-affecting GMP with physical exercise could have enhanced ability to target the multi-system decline often facing dementia patients.

## CONCLUDING REMARKS

Alzheimer’s disease (AD) is one of the most prevalent age-related inflictions among the elderly population, but there are currently no available therapeutics of any kind capable of preventing or curing this disease state. One potential approach is through the gut, but significant work is required to determine how such interventions can be implemented and the degree to which they can be efficacious. Probiotic-based interventions may be a good choice, provided there is an appropriate vessel through which systemic alterations can be administered. Though there is limited clinical evidence for the effectiveness of probiotics in dementia, there is a strong link between gut microbiome and brain function/dysfunction, including distinct microbial profiles in major depressive disorder (Zheng et al., 2016), autism spectrum disorders (Krajmalnik-Brown et al., 2015), schizophrenia (Shen et al., 2018) and microbiome-dependent resistance to seizure activity (Olson et al., 2018).

Thus, as outlined in this review, the utilization of genetically-modified probiotics (GMPs) may hold promise for AD-related impairments via oral delivery of therapeutic compounds. Specifically, the utilization of GMPs from lactic acid bacteria which itself has inherent benefits and these GMPs can be further utilized to deliver compounds such as Angiotensin (1–7) as there is strong evidence for the role of the RAS in AD (Wright and Harding, 2010). We look forward to seeing the continued development of work in this space in effort to identify efficacious interventions to combat the ever-growing prevalence of AD and other forms of dementia in the rapidly aging population.

## Figures and Tables

**TABLE 1 |:** Pre-clinical rodent Alzheimer’s disease model investigations involving RAS-affecting drugs and their main findings.

	Rodent model	Age/Sex	Intervention	Duration of intervention	Main findings
Torika et al. (2018)	5XAD transgenic mice (Tg6799)	2 months/unknown sex	Intranasal ARB (Candesartan)	8 weeks	Reduced hippocampal microglial activation and Aβ pathology
Trigiani et al. (2018)	APP mutation mice	3–4 months/male & female	Oral or subcutaneous osmotic mini-pump ARB (candesartan)	2–5 months	Lowered blood pressure, reduced neuroinflammation, increased dendritic arborization, and cellular proliferation but did not improve cognition or reduce Aβ pathology
Ongali et al. (2014)	APP mutation mice	15 months/male & female	Oral ARB (Losartan)	3 months	Losartan consolidates acquisition and recall memory, rescued cerebrovascular function; no reduction of Aβ pathology
Royea et al. (2017)	APP mutation mice	3 months	Subcutaneous osmotic mini-pump ARB (losartan)	3–4 months	Losartan improved memory retrieval, but not spatial learning, which was reversed by AT4R blockade. No alteration in Aβ pathology.
Mogi et al. (2008)	Intracerebroventricular Aβ_1–40_ injection Mice	2 months/male	Oral ARB (telmisartan or losartan)	2 weeks	Telmisartan and losartan prevented intracerebroventricular Aβ-induced cognitive impairment, but only telmisartan improved Aβ deposition
Tsukuda et al. (2009)	Intracerebroventricular Aβ_1–40_ injection mice	2 months/male	Oral ARB (telmisartan)	2 weeks	Telmisartan improved cognition function, decreased tumor necrosis factor-a, and reduced Aβ concentration
Takeda et al. (2009)	Intracerebroventricular Aβ_1–40_ injection mice & APP mutation mice	2 months/male	Oral ARB (olmesartan)	4–5 weeks	Olmesartan attenuated cerebrovascular dysfunction without reduction of Aβ pathology. It also improved cognition, prevented vascular dysregulation, and partially attenuated impaired hippocampal synaptic plasticity in other mice
Ferrington et al. (2011)	3xTgAD mice	3–4 months/male	Oral ARB (eprosartan or valsatan)	2 months	No change in Aβ or APP pathology
Ferrington et al. (2012)	3xTgAD Mice	9–10 months/male	Oral ARB (eprosartan)	6 months	No alteration in cognitive outcomes nor Aβ, tau, or APP levels
Wang et al. (2007)	APP mutated mice	6 months/female	Oral ARB (valsartan)	5 months	Reduced AD-type neuropathology and soluble extracellular oligomeric Aβ
Yamada et al. (2010)	Intracerebroventricular Aβ_25–35_ injection Mice	5–6 weeks/male	Oral ACEI (perindopril, imidapril or enalapril)	1 daily dose before testing	Only perindopril prevented working & long term memory deficits
Dong et al. (2011)	Intracerebroventricular Aβ_1–40_ injection Mice	Not specified/male	Oral ACEI (perindopril, imidapril, or enalapril)	7 days	Only perindopril prevented spatial memory impairment, prevented hippocampal microglial & astrocytic activation, and attenuated oxidative stress
Torika et al. (2016)	5XAD transgenic mice (Tg6799)	2 months/male	Intranasal ACEI (perindopril or captopril)	3.5–7 weeks	Attenuate AD-associated markers in cortex, reduced hippocampal, & cortical Aβ burden
Ferrington et al. (2011)	3xTgAD Mice	3–4 months/male	Oral ACEI (captopril)	2 months	No change in Aβ or APP pathology
Ferrington et al. (2012)	3xTgAD Mice	9–10 months/male	Oral ACEI (captopril)	6 months	No alteration in cognitive outcomes nor Aβ, tau, or APP levels
AbdAlla et al. (2013)	APP mutated mice	12 months	Oral ACEI (captopril or enalapril)	6 months	Slowed Aβ plaque development and Aβ-related neurodegeneration

3xTgAD, C57BL6 background mice with 3 AD-linked mutations; 5xAD, C57BL6 background mice with 5 AD-linked mutations; ACEI, Angiotensin-converting enzyme inhibitor; AD, Alzheimer’s disease; APP, Amyloid precursor protein; ARB, Angiotensin receptor blocker.

## Data Availability

The original contributions presented in the study are included in the article/supplementary material, further inquiries can be directed to the corresponding author/s.
